# Faecal Microbial and Metabolic Profiles Covary With Dietary Plant Composition in Tibetan Sheep Across Contrasting Grasslands

**DOI:** 10.1111/1758-2229.70443

**Published:** 2026-11-09

**Authors:** He Ding, Nan Liu, Linyao Chen, Zenghui Liu, Wentao Zhang, Fangyu Gao, Kenan Li, Hai Wang, Jianshuang Wu, Xiaoqing Zhang

**Affiliations:** ^1^ Institute of Grassland Research, Chinese Academy of Agricultural Sciences Hohhot China; ^2^ Tibet General Station of Animal Husbandry Lhasa China; ^3^ Institute of Environment and Sustainable Development in Agriculture Chinese Academy of Agricultural Sciences Beijing China

**Keywords:** dietary plant composition, faecal metabolomics, faecal microbiota, grazing ecology, Qinghai–Tibet plateau, Tibetan sheep

## Abstract

Alpine meadows (AM) and desert steppes (DS) are major grassland types in northern Qiangtang that support ecosystem functioning, livestock production and pastoral livelihoods. However, how Tibetan sheep coordinate dietary, microbial and metabolic responses across these contrasting forage environments remains unclear. We used vegetation surveys, organic matter intake (OMI) estimation, faecal DNA metabarcoding, 16S rRNA gene sequencing and untargeted metabolomics to compare female Tibetan sheep grazing AM or DS. The DS had lower forage biomass and concentrations of several nutrients, whereas DS sheep had higher OMI, lower dietary plant diversity and greater representation of Rosaceae (*p* < 0.05). Faecal bacterial communities differed between groups. Plant‐family composition, rather than dietary alpha diversity, was associated with bacterial variation; Rosaceae and Tamaricaceae showed the strongest associations (*r* ^2^ = 0.929 and 0.836, respectively; *p* = 0.001). Selected bacterial genera were associated with Rosaceae (Mantel *R* = 0.59, *p* = 0.001). Among 3819 detected metabolites, 1177 differed between groups and were mainly enriched in flavonoid‐ and phenylpropanoid‐related pathways (adjusted *p* < 0.05). These findings reveal coordinated associations among diet, faecal microbiota and metabolites and identify plant‐family composition as a key correlate of habitat‐related microbial and metabolic variation.

## Graphical abstract

Contrasting alpine meadow and desert steppe environments were associated with coordinated shifts in Tibetan sheep dietary plant composition, faecal bacterial communities and metabolites. Plant‐family composition was more strongly associated with microbial variation than dietary alpha diversity, while major metabolite differences involved flavonoid‐ and phenylpropanoid‐related pathways.

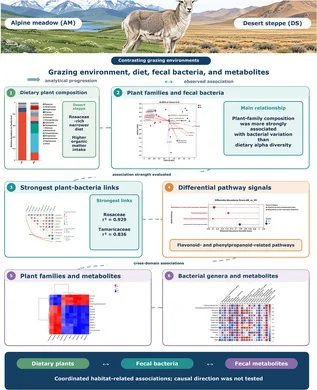

## 1 Introduction

The Qiangtang Plateau in the northern Qinghai–Tibet Plateau is a major alpine pastoral region of ecological and socioeconomic importance. It contains approximately 6.227 × 10^5^ km^2^ of natural grasslands, accounting for about 59% of the natural grassland area of the Tibet Autonomous Region (Liu et al. 2024). Alpine meadow and desert steppe are two widely distributed grassland types in northern Tibet (Yan et al. 2022) and differ in forage productivity and nutritional characteristics (Shi et al. 2013). These grasslands support soil and water conservation, carbon storage, biodiversity, livestock production and pastoral livelihoods. Tibetan sheep are a major livestock resource in the region and are central to local pastoral economies and culture (Liang et al. 2023). Spatial heterogeneity, seasonal variation, climate and grazing pressure alter the forage resources available to livestock; understanding how the dietary, microbial and metabolic characteristics of Tibetan sheep covary with contrasting forage environments is therefore important for animal production and grassland management.

Diet selection is a primary way in which herbivores respond to heterogeneous forage resources. On the Qinghai–Tibet Plateau, herbivores adjust dietary breadth and plant use with habitat heterogeneity, forage availability and grassland degradation (He et al. 2025; Ru et al. 2026; Wang et al. 2024). Faecal DNA metabarcoding of free‐grazing yaks has shown that seasonal turnover in consumed plants covaries with changes in the distal gut microbiota (Guo et al. 2021). In Tibetan sheep, rumen microbial structure and predicted function vary with plant phenology (Liu et al. 2020), while seasonal diet can explain distal gut microbial variation more strongly than host species in sympatric yaks and sheep (Wei et al. 2021). Together, these studies identify dietary context as an important correlate of gastrointestinal microbial structure. However, most work has focused on season, phenology, or broad feeding conditions rather than comparing the plant composition consumed by free‐grazing Tibetan sheep in contrasting natural grasslands.

Faecal DNA metabarcoding provides a non‐invasive, taxonomically resolved estimate of herbivore diet composition, although read abundance is only semi‐quantitative because it can be influenced by marker performance and differential digestion (Vallin et al. 2026). Faecal samples also provide a common matrix for integrating dietary plant DNA, bacterial profiles and metabolic end products. Multi‐omics studies in Tibetan sheep have reported concurrent microbial and metabolic differences under contrasting cold‐season feeding regimes (Wang, Zhang, et al. 2025) and across altitudes (Wang, Liu, et al. 2025). Related studies have described livestock‐specific rumen microbiome–metabolome profiles (Omondi et al. 2024) and associations between diet and gut microbiota in sympatric plateau herbivores (Zhang et al. 2025). Nevertheless, it remains unclear whether plant‐family identity or dietary diversity is more strongly associated with faecal bacterial variation in free‐grazing Tibetan sheep and whether the same dietary differences covary with faecal metabolites.

Here, we compared Tibetan sheep grazing alpine meadow and desert steppe area in northern Qiangtang. We examined whether differences in local forage resources and faecal‐reconstructed diet composition were accompanied by differences in faecal bacterial communities and metabolite profiles. We hypothesized that the two forage environments would be associated with distinct dietary compositions and that plant‐family identity, rather than dietary diversity alone, would show stronger associations with bacterial and metabolic variation. By integrating vegetation measurements, organic matter intake, faecal DNA metabarcoding, 16S rRNA gene sequencing and untargeted metabolomics, this study evaluates coordinated diet–microbiota–metabolite associations without assuming a causal pathway.

## 2 Materials and Methods

### 2.1 Trial Location

The experiment was conducted in alpine meadow (AM) (87°16′ E, 31°30′ N) and desert steppe (DS) (87°19′ E, 32°10′ N) with an average altitude approximately 4700 m above sea level in Nima County, Naqu City, Tibet Autonomous Region of China, in July 2025 (Figure 1). The AM was dominated by *Blysmus sinocompressus* Tang and Wang, *Hedysarum tibeticum* (Benth.) B. H. Choi and H. Ohashi, *Potentilla multifida* L., *Lancea tibetica* Hook.f. and Thomson and *Astragalus arnoldii* Hemsl. and H. Pearson, while the DS by *Carex parvula* O. Yano, *Potentilla cuneata* Wall. ex Lehm., *Leontopodium ochroleucum* Beauv., *Rhodiola heterolata* (Frod.) S. H. Fu and *Potentilla bifurca* L.

**FIGURE 1:**
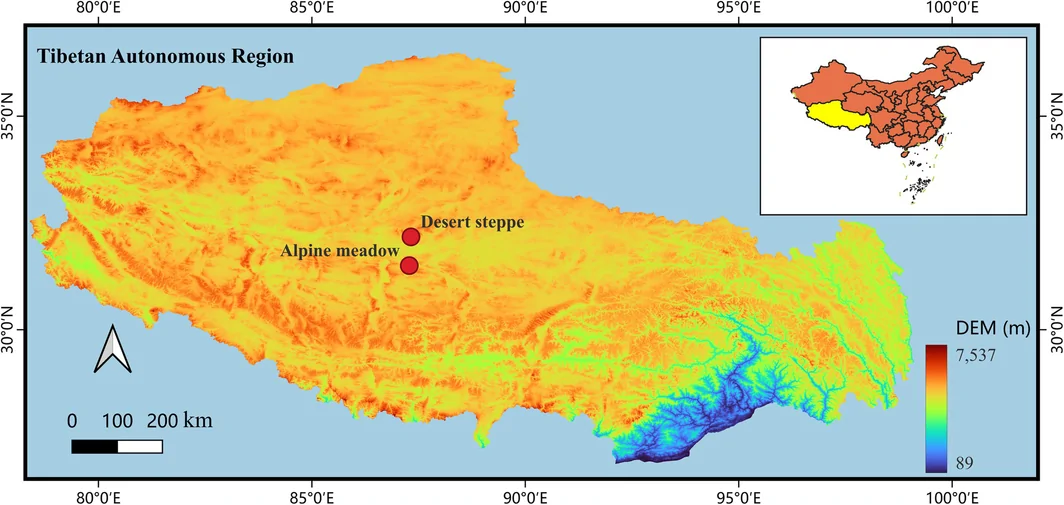
Sampling map of Tibetan sheep in Nima County, Naqu City of the Qinghai–Tibet Plateau. Two different grassland types including alpine meadow and desert steppe (red circles in the map) were displayed in the sampling map; nine Tibetan sheep were randomly selected from each type of grassland. The map was created with version 2.18 of QGIS software.

### 2.2 Experimental Design, Animal Management and Sampling

In the present research, the Animal Ethics Committee of the Chinese Academy of Agricultural Sciences approved our animal welfare and experimental procedure (No. AEC‐CAAS‐202501ZY0042). Two sheep flocks with approximately 200 sheep in each flock in AM and DS above mentioned, respectively, were selected. The local flocks had continuously grazed the same respective grassland areas from May to October 2025. Throughout the grazing season, both flocks were managed under the same daily schedule and general husbandry practices. The sheep grazed freely on their respective natural grasslands from 08:00 to 20:00 and were kept in pens from 20:00 to 08:00. No supplementary concentrate or harvested forage was provided, and drinking water was available ad libitum. The faecal sampling experiment was conducted in late July 2025, after the animals had been exposed to their respective grazing environments for approximately 3 months. Female Tibetan sheep with a similar body weight of 25.33 ± 1.03 kg (Mean ± SEM) and at the age of 2 years old from (nine sheep/flock, totally 18 sheep selected) these grazing grassland types were randomly selected. Faecal samples were obtained from the rectum of selected sheep with nitrile gloves on three consecutive days; equal amounts of sheep faeces collected over the 3 days were thoroughly blended and placed into the cryopreservation tubes, and quickly stored in liquid nitrogen for transport to the laboratory, then subsequently stored at −80°C in the laboratory for further analysis.

Six sheep of each group within the experimental sheep abovementioned were randomly selected using titanium dioxide as an exogenous indicator to predict the organic matter intake (OMI). Each sheep was fed three capsules with 0.5 g titanium dioxide of each capsule before grazing in the morning. The adaptation period was 5 days, following 3 days for faecal samples collection. After grazing, the faecal samples were collected through the rectum of sheep. At the end of feeding experiment, all faecal samples were mixed and dried at 65°C using an oven to constant weight. The crude protein in faeces was determined by the Kjeldahl method, and concentration of titanium by an inductively coupled plasma mass spectrometer (ICP‐MS, iCAP RQ, Thermo Fisher Scientific Inc., Waltham, MA, USA) and then converted to that of titanium dioxide. The formula for calculating organic matter intake based on the concentration of titanium dioxide was detailly described by Glindemann et al. (2009) and Wang et al. (2009).

### 2.3 Vegetation Survey and Laboratory Analyses

During the same period of sheep faecal sampling in the late July 2025, above‐ground biomass was collected from six randomly selected quadrats (50 cm × 50 cm) within a grazing area for sheep in the AM and DS areas, dried in an oven at 65°C until a stable weight was reached and categorized into four functional groups: forbs, legumes, poaceae and cyperaceae. Crude protein (CP) was determined by the Kjeldahl method (AOAC Official Method 2001.11) and calculated as nitrogen × 6.25. Ether extract (EE) was measured by solvent extraction (AOAC Official Method 920.39), ash by furnace incineration (AOAC Official Method 942.05) and crude fibre (CF) was determined according to AOAC Official Method 978.10. Neutral detergent fibre (NDF) was determined as amylase‐treated NDF using heat‐stable α‐amylase according to AOAC Official Method 2002.04, whereas acid detergent fibre (ADF) and lignin were analysed according to AOAC Official Method 973.18.

### 2.4 Dietary Sequencing and Annotation

DNA of sheep faecal samples was extracted using E.Z.N.A. soil DNA kit (Omega Bio‐tek, Norcross, GA, US) according to the manufacture instruction. Agarose gel electrophoresis was used to evaluate the quality of extracted genomic DNA. The NanoDrop2000 spectrophotometer (Thermo Scientific, USA) was employed to determine the DNA samples' concentration and purity. The trnL intron and trnL‐trnF intergenic spacer were amplified using the universal chloroplast primers trnL‐F (5′‐CGAAATCGGTAGACGCTACG‐3′) and trnR (5′‐CCATTGAGTCTCTGCACCTATC‐3′) (Taberlet et al. 1991). PCR amplification was conducted under the following cycling conditions: start with denaturation at 95°C for 3 min, proceed with 35 cycles of denaturation at 94°C for 25 s, annealing at 56°C for 25 s and extension at 72°C for 10 s, then a single extension at 72°C for 4 min and finish at 10°C (ABI GeneAmp Model 9700). The PCR product was isolated from a 2% agarose gel, purified with the PCR Clean‐Up Kit from YuHua in Shanghai, China, as per the manufacturer's instructions, and measured using Qubit 4.0 from Thermo Fisher Scientific, USA. Equimolar amounts of purified amplicons were combined and sequenced using paired‐end technology on an Illumina Nextseq2000 platform, following the standard procedures of Majorbio Bio‐Pharm Technology Co. Ltd. in Shanghai, China.

After demultiplexing, the resulting sequences were quality filtered with fastp (0.23.4) (Magoc and Salzberg 2011) and merged with FLASH (1.2.11) (Chen et al. 2018). Then the high‐quality sequences were de‐noised using DADA2 (Edgar 2013) plugin in the Qiime2 (version 2024) (Wang et al. 2007) pipeline with recommended parameters, which obtains single nucleotide resolution based on error profiles within samples. DADA2 denoised sequences are usually called amplicon sequence variants (ASVs). To minimize the effects of sequencing depth on alpha and beta diversity measure, the number of sequence from each sample was rarefied to 20,000, which still yielded an average Good's coverage of 99.98%. Taxonomic assignment of ASVs was performed using the Blast consensus taxonomy classifier implemented in Qiime2 and the SILVA 16S rRNA database (v138).

Mothur v1.30.1 (Schloss et al. 2009) was employed to calculate rarefaction curves and alpha diversity indices, including Chao richness, Shannon index, Pielou_e and Good's coverage, based on the ASVs information. To characterize the relationships in diet composition among faecal samples, Bray–Curtis distances were calculated and used for average linkage hierarchical clustering, with dendrograms generated using the Vegan package in R (version 3.3.1). The stacked bar chart of diet profiles was generated in Python (version 2.7) to show the top 15 families according to the relative abundance. Differential abundances of diet species were determined by the Wilcoxon rank‐sum test with false discovery rate (FDR, < 0.05) correction, and bar plots of top 10 families were generated in stat of the R software (version 3.3.1). The ‘randomForest’ package in R software was utilized to conduct a random forest analysis, identifying the Top 10 crucial plant families for sheep grazing across various grasslands.

### 2.5 Microbiota Composition of Sheep Faecal Samples

DNA was also extracted from faecal samples using the same DNA extraction process as plant DNA metabarcoding above mentioned. The hypervariable region V3–V4 of the bacterial 16S rRNA gene were amplified with primer pairs 338F (5′‐ACTCCTACGGGAGGCAGCAG‐3′) and 806R(5′‐GGACTACHVGGGTWTCTAAT‐3′) (Liu et al. 2016) byT100 Thermal Cycler PCR thermocycler (BIO‐RAD, USA). PCR amplification cycling conditions were as follows: initial denaturation at 95°C for 3 min, followed by 27 cycles of denaturing at 95°C for 30 s, annealing at 53°C for 30 s and extension at 72°C for 45 s and single extension at 72°C for 10 min, and end at 10°C. The PCR product was extracted from 2% agarose gel and purified using the PCR Clean‐Up Kit (YuHua, Shanghai, China) according to manufacturer's instructions and quantified using Qubit 4.0 (Thermo Fisher Scientific, USA). Illumina sequencing, amplicon sequence processing and analysis were also the same as the process of dietary plants analysis.

Mothur v1.30.1 (Schloss et al. 2009) was employed to calculate rarefaction curves and alpha diversity indices, including Chao richness, Shannon index, Pielou_e and Good's coverage, based on the ASVs information. The Bray‐Curtis distance matrix was employed for similarity analysis (ANOSIM) with 999 permutations, and PCoA was used to visualize the microbial community composition in sheep faeces from different grasslands. Distance‐based redundancy analysis (db‐RDA) was conducted to relate the diversity indexes and important plant families with the faecal microbiome community at genus level based on Bray‐Curtis distance matrice (using the R software of version 3.3.1 with vegan of version 2.4.3). A linear discriminant analysis (LDA) effect size (LEfSe) analysis software (http://galaxy.biobakery.org/) was used to find the top 10 essential microbiome of each group according to LDA scores. The R package ‘ggcor’ was used for the Mantel test based on Spearman's correlation test between dietary essential plant families and genus. The biological functions of faecal bacteria were predicted using PICRUSt2 (Douglas et al. 2020), and the differential relative abundance comparison of pathways was analysed with the student's test, and the false discovery rate (FDR) was applied to correct for multiple comparisons. Correlation analysis of genera identified by LEfSe and differential pathways was conducted using the spearman correlation test, and significant correlation was considered at *p* < 0.05 and coefficient |*r*| > 0.8.

### 2.6 Non‐Targeted Metabolome Analysis

#### 2.6.1 Metabolite Separation in Sheep Faecal Samples

Metabolomic analysis of sheep faecal samples from the AM group (*n* = 9) and the DS group (*n* = 9) was completed with the ultrahigh‐performance liquid chromatography quadrupole‐time‐of‐flight mass spectrometry (UPLC‐TripleTOF) system (AB SCIEX). An appropriate number of faecal samples was transferred to a high‐throughput tissue homogenizer, and 400 μL of cold 80% methanol solution was added for disruption. After the samples were homogenized, they were thoroughly vortexed and subjected to ultrasonic extraction on ice thrice (10 min each). After ultrasonic treatment, samples underwent 30 min of incubation under −20°C before centrifugation (13,000*g*, 15 min, 4°C). Next, supernatants were carefully added to vials of the LC–MS autosampler.

#### 2.6.2 LC–MS Measurement Conditions

Chromatographic separation of the metabolites in the sheep faecal samples was conducted using the BEH C18 column (100 mm × 2.1 mm, 1.7 μm; Waters). Mobile phases included phase A, ultrapure water that contained 0.1% formic acid, alongside Phase B, an acetonitrile‐isopropanol mixture (1:1, v/v) that contained 0.1% formic acid. Gradient elution included: 0–3 min, linear elevation of B from 0% to 20%; 3–9 min, B elevation from 20% to 60%; 9–11 min, further B elevation from 60% to 100%, followed by a plateau at 100% B for 2.5 min. The solvent composition rapidly returned from 100% to 0% B during 13.5–13.6 min, followed by maintenance at 0% B for 2.4 min. Column temperature, injection volume and flow rate were 40°C, 20 μL and 0.40 mL/min. MS signal detection was completed in positive/negative‐ion mode to identify the eluted material.

#### 2.6.3 Comparison of Differentially Abundant Metabolites

After raw data were imported into Progenesis QI software (Waters Corporation, Milford, USA), the baseline was filtered, then the peaks were identified, integrated, corrected for retention time and aligned. Metabolites were subsequently identified by matching mass spectrometry information with the Human Metabolome Database (HMDB) and the Kyoto Encyclopedia of Genes and Genomes (KEGG), generating the data matrix used in further analyses. Unpaired Student's *t*‐test and partial least squares discriminant analysis (PLS‐DA) were completed with ropls (R packages, Version 1.6.2). Volcano plots were produced using the pandas package (version 0.23.4) in Python, which identified significantly differentially abundant metabolites in two groups at VIP > 1.0 and *p* < 0.05 thresholds in line with PLS‐DA and Student's *t*‐test results. Metabolic enrichment and pathway analysis, based on the KEGG database (http://www.genome.jp/kegg/), were used to map the differential metabolites of the two groups into their biochemical pathways. The method for enrichment involved a hypergeometric test, topology analysis was based on relative‐betweenness centrality and *p*‐values were adjusted using the Benjamini‐Hochberg (BH) correction for multiple tests. Significant enrichment was observed in pathways with an adjusted *p*‐value < 0.05. KEGG analysis enrichment results were represented as a differential abundance score plot of pathways and a network diagram reflecting correspondence between varied metabolites and pathways. VIP bar plots of the top 15 metabolites in the database related to significant pathways identified differentially abundant metabolites with VIP > 1.0 that were created with Python software (scipy packages, Version 1.0.0). The spearman correlation analysis was done between the top 15 metabolites and microbiome (essential genera determined by LEfSe) or plants (important families identified by the random forest analysis), and results with *p* < 0.05 meaning significant relationship.

### 2.7 Statistical Analysis

The unpaired Student's *t*‐test (SAS version 9.2; SAS Institute Inc., Cary, NC, USA) was used for two‐group comparisons of proximate composition (CP, EE, ash, CF, NDF, ADF and lignin), plant community diversity (Chao and Simpson indices), and functional group biomass (legume, Cyperaceae, forbs, Poaceae and total). Results are expressed as means ± standard error of the mean, with statistical significance set at *p* < 0.05. Violin plots for plant community diversity and bar plots for functional group biomass were created using Prism version 9.3.1 (GraphPad Software, San Diego, CA, USA).

## 3 Results

### 3.1 Biomass of Functional Groups, Diversity and Proximate Composition of Plant Community

For diversity of plant community, Chao index in the DS group, were significantly lower than those in the AM group (*p* < 0.05, Figure 2A), while there was no significance for the Simpson index within groups (Figure 2B). As for biomass of functional groups, the above biomass for both the AM and the DS groups, could be classified into legume, poaceae, cyperaceae and forbs. Plants in the AM group were mainly composed of forbs (18.39%), poaceae (10.59%), legumes (9.73%) and for the DS group, were Cyperaceae (11.11%) and forbs (4.34%) (Figure 2C). Biomass of legume (Figure 2D, *p* < 0.001), forbs (Figure 2F, *p* = 0.007), poaceae (Figure 2G, *p* < 0.001) and total biomass (Figure 2H, *p* = 0.020) in the AM group was significantly higher than those in the DS group, while the contrast result was suitable for cyperaceae (Figure 2E, *p* < 0.001). As for proximate composition of plant community, CP, EE, Ash, CF, lignin (Table 1), in the DS group, were significantly lower than those in the AM group (*p* < 0.05).

**FIGURE 2:**
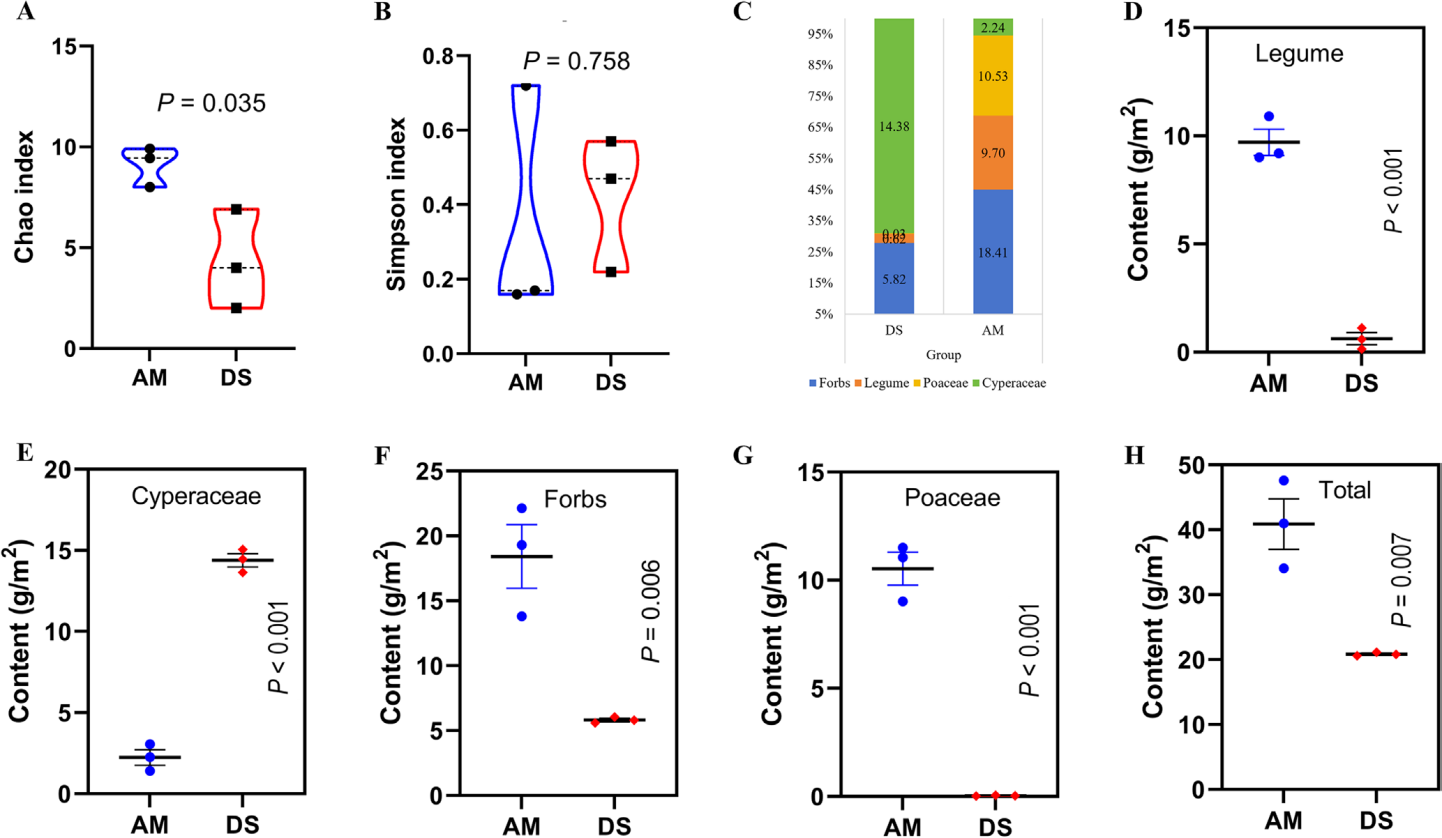
Diversity of plant community and biomass of functional groups in alpine meadow (AM) and desert steppe (DS) grasslands. (A) Chao index, and (B) Simpson index of plant community; (C) Percentage stacked chart of plant composition; Content of legume (D), Cyperaceae (E), forbs (F), poaceae (G) and total biomass of functional groups (H), respectively.

**TABLE 1:** Comparison of proximate composition of plant community of the AM and the DS grasslands (Mean ± SEM).

Grassland type	Proximate composition (g/m^2^)						
	CP	EE	Ash	CF	NDF	ADF	Lignin
AM	4.98 ± 0.44	1.45 ± 0.08	5.09 ± 0.72	11.62 ± 1.08	14.97 ± 1.22	8.74 ± 0.48	4.75 ± 0.25
DS	2.00 ± 0.35	0.31 ± 0.06	1.57 ± 0.32	5.74 ± 1.04	7.59 ± 2.58	3.79 ± 1.32	1.07 ± 0.36
*p*‐value	0.013	0.001	0.014	0.033	0.123	0.066	0.005

Abbreviations: ADF, acid detergent fibre; AM, alpine meadow; CF, crude fibre; CP, crude protein; DS, desert steppe; EE, ether extract; NDF, neutral detergent fibre.

### 3.2 Organic Matter Intake and Dietary Composition of Sheep Within Groups

The OMI of sheep in the DS group was remarkably higher compared to that in the AM group (*p* < 0.05). At family level, the value of coverage index was all close to 1 between AM and DS groups, and chao, pielou_e and shannon indexes at family (Figure 3A–D) level in the DS group were all significantly lower than those in the AM group (*p* < 0.05). Hierarchical clustering analysis showed that the samples of each group were better clustered together at family (Figure 3E) level. The dominant families of the DS group were Rosaceae, Poaceae and Asteraceae, while for the AM group were Tamaricaceae, Polygonaceae and Rosaceae (Figure 3F). As the comparison of the relative abundance (RA) of families, top 10 differential families of shared species within groups according to the RA were selected. RA of the Rosaceae was significantly higher in the DS group compared to that in the AM group (Figure 3G, *p*_adjust_ < 0.05). However, Tamaricaceae, Polygonaceae, Asteraceae, Fabaceae, Cyperaceae, Plantaginaceae, Mazaceae, Brassicaceae and Caryophyllaceae were higher in the AM group (Figure 3G, *p*_adjust_ < 0.05). Top 10 essential families for each group were screened out by the random forest analysis (Figure 3H,I for the DS and the AM groups, respectively), and top 4 important plant families were the same for both groups; they were composed of Polygonaceae, Rosaceae, Tamaricaceae and Cyperaceae. However, the other 6 essential families were Mazaceae, Fabaceae, Boraginaceae, Caryophyllaceae, Orobanchaceae and Plantaginaceae for the DS group, while Boraginaceae, Mazaceae, Fabaceae, Plantaginaceae, Orobanchaceae and Caryophyllaceae were for the AM group.

**FIGURE 3:**
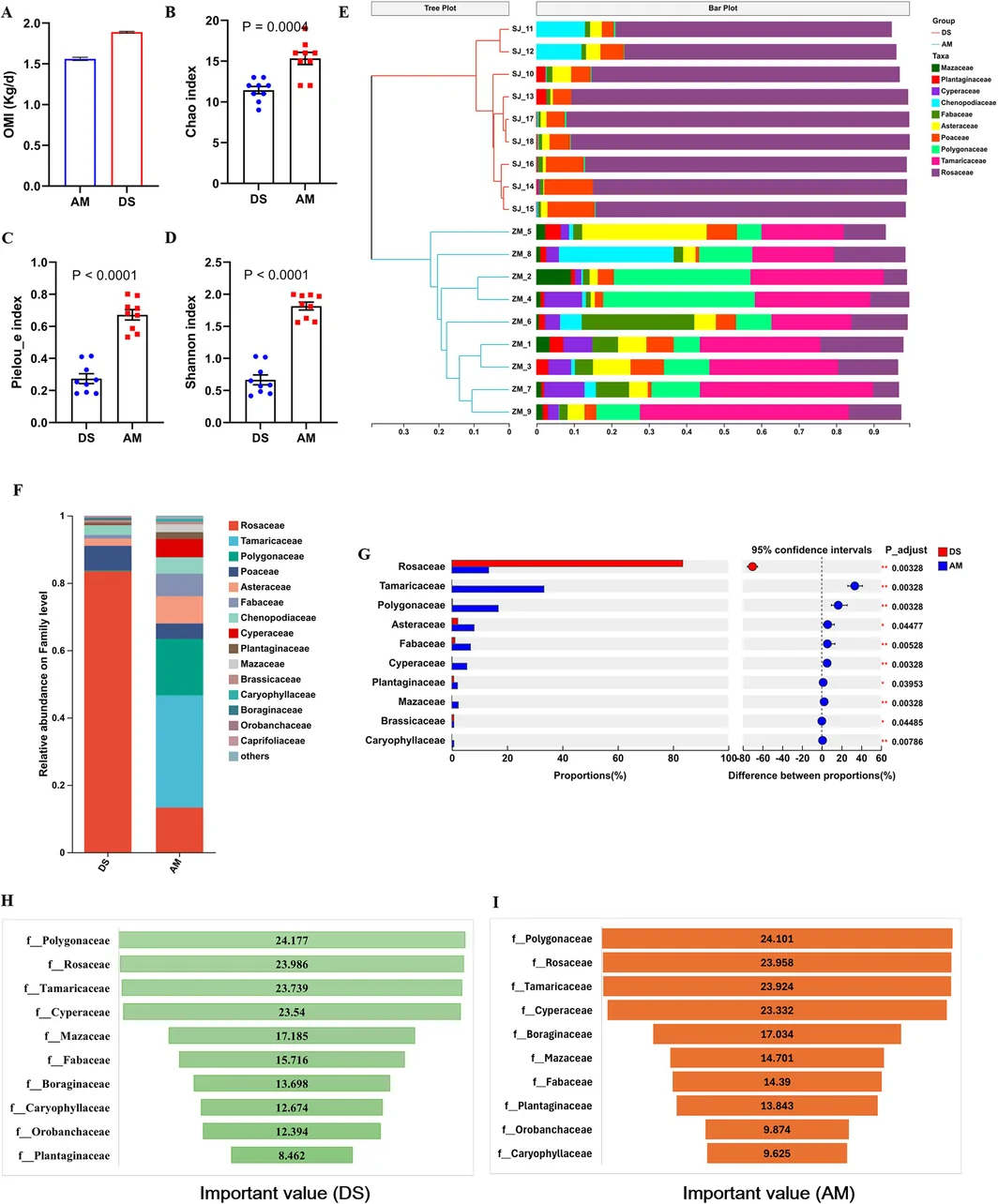
Foraging diet composition and differential comparison of family level. (A–D) The Organic matter intake (OMI) predicted by titanium dioxide and alpha diversity indexes of chao, pielou_e, and shannon, respectively. (E) Hierarchical clustering analysis based on bray_curtis with the method of average. (F) Community bar plot analysis of families of foraging diet. (G) Differential analysis of grass families. (H and I) Random Forest analysis showing the importance of dietary composition of the DS and the AM groups. AM, alpine meadow; DS, desert steppe.

### 3.3 Comparison of the Microbiome Compositions in Sheep Faeces Between Groups

The PCoA revealed notable differences in the microbial composition between the DS group and the AM group (Figure 4A). Based on db‐RDA (Figure 4B), the correlations of vegetation variables with CAP1 and CAP2 axes, explanatory power (*r* ^2^) and significance were analysed (detailed results displayed in Table S1). All alpha diversity indices, including Chao, Shannon and Pielou_e, showed low explanation and no significant correlations with microbial community variation (*p* > 0.05). However, several plant families strongly correlated with the CAP axes and significantly shaped microbial community structure. Boraginaceae was positively correlated with CAP1 (0.3468) and negatively with CAP2 (−0.9379, *r* ^2^ = 0.6319, *p* = 0.002). Rosaceae exhibited a strong positive correlation with CAP1 (0.9662), while Tamaricaceae showed a strong negative correlation with CAP1 (−0.9642); both had high explanatory values (r2 = 0.929 and 0.8358, respectively, *p* = 0.001). Significant families (*p* < 0.05) mainly presented negative CAP1 coefficients, including Caryophyllaceae (−0.9774), Cyperaceae (−0.8978) and Polygonaceae (−0.8678). Overall, plant family composition rather than plant alpha diversity primarily drove the differentiation of microbial communities. The top 10 essential genus biomarkers for each group were identified by the LEfSe analysis based on LDA values rank and *p* < 0.05, and *g__NK4A214_group*, *g__Flavonifractor*, *g__Ruminococcus*, *g__norank_f__Muribaculaceae*, *g__norank_f__p‐251‐o5*, *g__Berryella*, *g__norank_f__Anaerovoracaceae*, *g__Roseburia*, *g__Lachnospiraceae_UCG‐010*, and *g__norank_f__Bacteroidales_RF16_group* were important biomarkers for the DS group, while *g__Prevotellaceae_UCG‐004*, *g__Alistipes*, *g__Candidatus_Soleaferrea*, *g__norank_f__Peptococcaceae*, *g__[Eubacterium]_oxidoreducens_group*, *g__Clostridium*, *g__norank_o__Izemoplasmatales*, *g__Dielma*, *g__norank_o__Rickettsiales*, and *g__Adlercreutzia* for the AM group (Figure 4C). The Mantel‐test displayed the microbiome (the selected 20 genera by LEfSe) in sheep faecal samples were positively correlated with Boraginaceae (*R* = 0.46, *p* = 0.001), Rosaceae (*R* = 0.59, *p* = 0.001), and Tamaricaceae (*R* = 0.44, *p* = 0.001) (Figure 4D).

**FIGURE 4:**
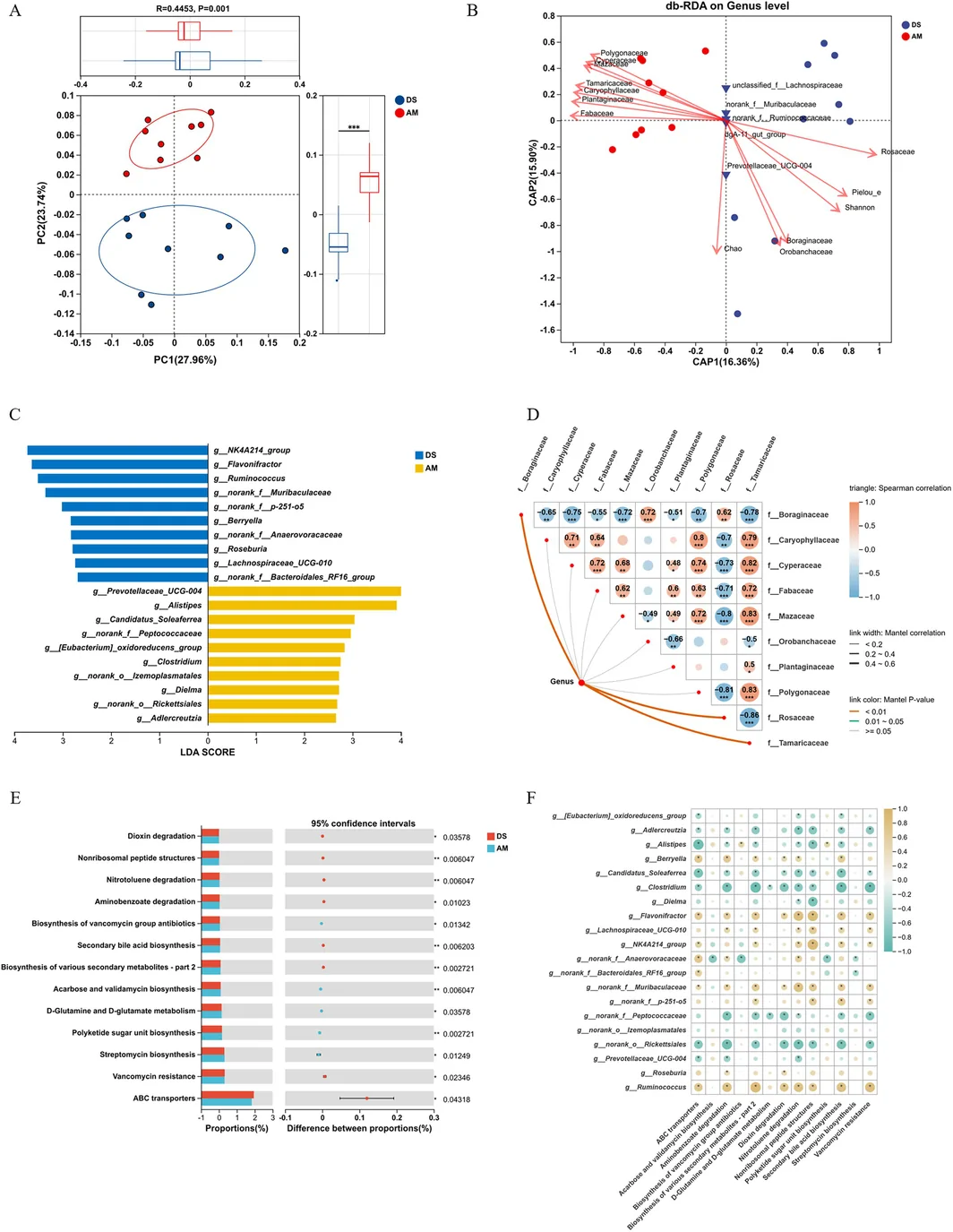
Comparison of the microbiome compositions in sheep faeces between groups. (A) Relative abundance of bacteria on phylum level. (B) The db‐RDA analysis to evaluate the influence of diversity and composition of vegetation in diets on faecal microbial composition. (C) The LEfSe analysis to select top 10 essential genera to AM and DS groups, respectively. (D) Mantel test between genus classification of differential genera with selected plant families. (E) Relative abundance of differential KEGG pathways. (F) Correlation of faecal genera and varied pathways.

Thirteen differential pathways between the DS and the AM groups were selected with *p*‐adjust < 0.05, and the relative abundance of Biosynthesis of vancomycin group antibiotics, Acarbose and validamycin biosynthesis, d‐Glutamine and d‐glutamate metabolism, Polyketide sugar unit biosynthesis and Streptomycin biosynthesis was increased in the AM group (*p*‐adjust < 0.05, Figure 4E), while the abundance of ABC transporters, Acarbose and validamycin biosynthesis, Aminobenzoate degradation, Biosynthesis of various secondary metabolites—part 2, Dioxin degradation, Nitrotoluene degradation, Nonribosomal peptide structures, Secondary bile acid biosynthesis and Vancomycin resistance was increased in the DS group (*p*‐adjust < 0.05, Figure 4E). Spearman correlation analysis showed that *g__Alistipes* exhibited a strong negative correlation with ABC transporters (*r* = −0.862, *p* < 0.05); *g__Clostridium* was strongly negatively associated with three metabolic pathways, including Biosynthesis of various secondary metabolites—part 2 (*r* = −0.876, *p* < 0.05), Secondary bile acid biosynthesis (*r* = −0.865, *p* < 0.05) and Vancomycin resistance (*r* = −0.874, *p* < 0.05); *g__norank_o__Rickettsiales* showed significant negative correlations with Aminobenzoate degradation (*r* = −0.837, *p* < 0.05) and Nitrotoluene degradation (*r* = −0.813, *p* < 0.05); *g__Flavonifractor* was positively correlated with Nitrotoluene degradation (*r* = 0.814, *p* < 0.05) and Nonribosomal peptide structures (*r* = 0.859, *p* < 0.05); *g__NK4A214_group* displayed the strongest positive correlation with Nonribosomal peptide structures (*r* = 0.878, *p* < 0.05); *g__Ruminococcus* was positively associated with Biosynthesis of various secondary metabolites—part 2 (*r* = 0.868, *p* < 0.05) and Vancomycin resistance (*r* = 0.827, *p* < 0.05) (Figure 4F).

### 3.4 Metabolomics Analysis of Sheep Faeces Between Groups

The total number of identified metabolites was 3819, and 2498 metabolites among them could be classified according to HMDB database. The top 5 classifications were Lipids and lipid‐like molecules, Organic acids and derivatives, Organoheterocyclic compounds, Benzenoids and Phenylpropanoids and polyketides according to the number of metabolites of each category (Figure 5A). PLS‐DA plots showed there was distinct separation of metabolites profile within groups, indicating significant differences existing between groups (Figure 5B). Among the total identified metabolites, 624 metabolites were up‐regulated, while 553 down‐regulated (*p* < 0.05, VIP > 1, FC > 1, Figure 5C). Pathways of flavone and flavonol biosynthesis, flavonoid biosynthesis, biosynthesis of phenylpropanoids and biosynthesis of various plant secondary metabolites were significantly down‐regulated in the AM group after differential metabolites being annotated by using KEGG database (*p*‐adjust < 0.05, Figure 5D). Network of differential pathways and corresponding metabolites was shown in Figure 5E. The top 15 metabolites taking part in varied pathways according to VIP values were selected, and Phlorizin, Apigenin, 3‐(2‐Hydroxyphenyl)Propanoic Acid and Ferulic Acid were significantly up‐regulated, while Sakuranetin, Hordatine A, Ppa, Matairesinol, Angelicin, 2′‐Deoxymugineic Acid, Gallic Acid, Afzelechin, Kaempferol, Cynaroside and Trifolin were significantly down‐regulated in the AM group compared to those in the DS group (*p* < 0.05, Figure 5F).

**FIGURE 5:**
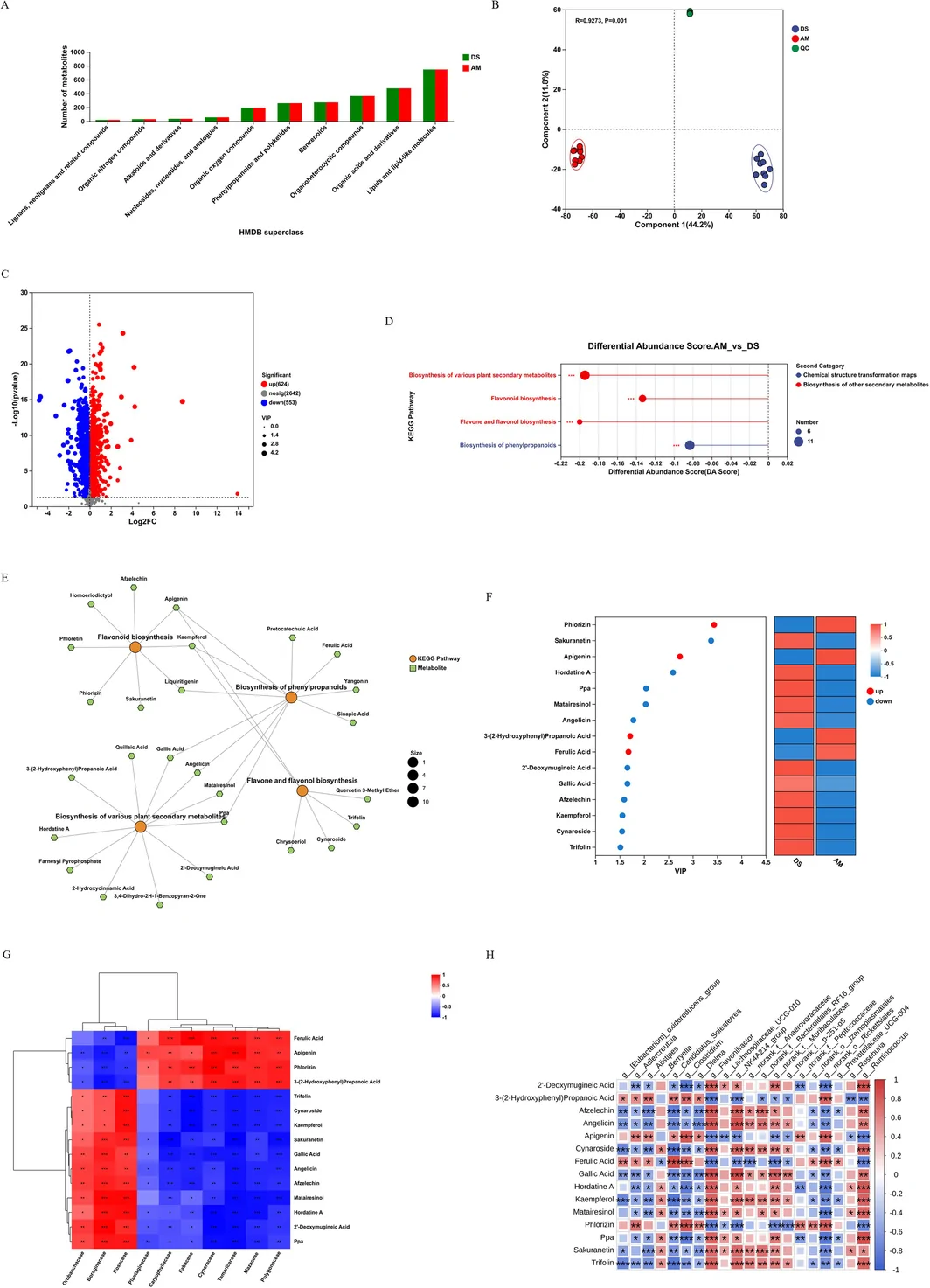
Untargeted LC–MS metabolomics analysis of sheep faeces between groups. (A) Bar chart of identified metabolites classification. (B) PLS‐DA analyses of metabolic profiling. (C) Volcano map of differential metabolites. (D) Abundance score plot of differential KEGG pathways. (E) Network of significant pathways and corresponding metabolites in this study. (F) The VIP score plots of the top 15 metabolites taking part in the differential pathways. (G) The correlation heatmap between essential plant families and top 15 metabolites. (H) The correlation heatmap between crucial faecal genera and top 15 metabolites.

Orobanchaceae, Boraginaceae and Rosaceae were mainly positively correlated with metabolites, whereas Caryophyllaceae, Fabaceae, Cyperaceae, Tamaricaceae, Mazaceae and Polygonaceae were predominantly negatively correlated. Among the strongest associations, ferulic acid was positively correlated with Fabaceae (*r* = 0.868, *p* < 0.001), while sakuranetin was negatively correlated with Tamaricaceae (*r* = −0.863, *p* < 0.001). In addition, flavonoid‐ and phenolic‐related metabolites, including cynaroside, kaempferol and matairesinol, showed consistent positive correlations with Rosaceae or Boraginaceae but negative correlations with Fabaceae, Tamaricaceae or Mazaceae (Figure 5G). Correlation analysis revealed clear clustered association patterns between differential bacterial genera and differential metabolites (Figure 5H). Overall, *Flavonifractor*, *NK4A214_group*, *Ruminococcus*, *norank_f__Muribaculaceae* and *norank_o__Rickettsiales* were mainly positively correlated with Afzelechin, Angelicin, Cynaroside, Kaempferol, Gallic acid, Ppa and Trifolin, but negatively correlated with 3‐(2‐Hydroxyphenyl)propanoic acid, Apigenin, Ferulic acid and Phlorizin; in contrast, *Clostridium*, *Candidatus_Soleaferrea*, *Alistipes*, *Adlercreutzia* and *Dielma* showed the opposite correlation pattern. Notably, *Candidatus_Soleaferrea* showed the strongest positive correlation with Ferulic acid (*r* = 0.871, *p* < 0.001), while it was strongly negatively correlated with Cynaroside and Kaempferol (*r* = −0.852, *p* < 0.001). Meanwhile, *Ruminococcus* was significantly positively correlated with Ppa (*r* = 0.868, *p* < 0.001) and Hordatine A (*r* = 0.827, *p* < 0.001) but negatively correlated with Apigenin (*r* = −0.794, *p* < 0.001) and Ferulic acid (*r* = −0.767, *p* < 0.001). Overall, these results suggest that shifts in faecal microbial composition were closely associated with the coordinated variation of phenolic acids, flavonoids and their derivative metabolites.

## 4 Discussion

The contrasting forage environments were accompanied by coordinated differences in dietary composition, faecal bacterial communities and faecal metabolite profiles. The stronger association of bacterial variation with plant‐family composition than with dietary alpha diversity, together with differences in flavonoid‐ and phenylpropanoid‐related metabolites, suggests that the nutritional and phytochemical characteristics of consumed plants may be more relevant to microbial and metabolic differentiation than dietary breadth alone. However, because dietary composition, faecal microbiota and metabolites were assessed concurrently in this observational study, the observed relationships should be interpreted as coordinated associations rather than evidence of a causal pathway.

### 4.1 Forage Constraints and Dietary Adjustment

Alpine meadows on the Tibetan Plateau generally support greater forage yield and higher overall forage quality than steppe‐like or desert grasslands because of differences in productivity and plant functional‐group composition (Shi et al. 2013; Wu et al. 2025). Against this background, the higher OMI observed in DS sheep is consistent with compensatory intake, whereby animals consume more forage when nutrient concentrations or harvestable biomass are limited. Comparable dietary adjustment has been reported for plateau herbivores exposed to resource constraints (He et al. 2025), and biomass–quality trade‐offs among plant functional groups further influence nutrient supply in Tibetan grasslands (Qin et al. 2025). Nevertheless, higher OMI does not demonstrate complete nutritional compensation, because nutrient supply depends on both intake and digestible nutrient concentration. Grazing behaviour, digestibility and nutrient balance would need to be measured to test this mechanism directly.

The combination of higher OMI and lower dietary diversity suggests that DS sheep obtained more material from a relatively restricted set of plants rather than broadening their diet. Rosaceae, including *Potentilla* species common in the studied DS area, may therefore constitute an important local dietary resource. Studies of plateau *Potentilla* document marked chemical diversity, including fatty acids, flavonoids and other phenolic compounds (Luan et al. 2022; Gao, Xing, et al. 2025; Li et al. 2024), providing a plausible link between botanical composition and downstream microbial and metabolic patterns. Previous studies similarly show that plant use by plateau herbivores varies with forage availability, nutrient traits, season and host species (Wang et al. 2024; Hou et al. 2026; Zhang et al. 2025). However, faecal DNA read abundance is affected by marker performance and differential digestion (Vallin et al. 2026). The greater representation of Rosaceae should therefore be interpreted as habitat‐related dietary composition arising from availability and selection together, not as proof of active preference for that family.

### 4.2 Plant‐Family Composition and Faecal Bacterial Differentiation

Plant‐family composition was more strongly associated with faecal bacterial variation than dietary richness or evenness. This distinction is biologically plausible because alpha‐diversity indices summarize the number and distribution of taxa but not their nutritional or phytochemical identities. Plant families differ in fermentable carbohydrates, structural fibre, nitrogen, minerals and secondary metabolites; these substrates can favour microorganisms with different degradative or tolerance traits, while phenolics and tannins may inhibit susceptible taxa or provide substrates for organisms capable of transforming aromatic compounds (Tedeschi et al. 2021; Santhiravel et al. 2022). The associations of Rosaceae and Tamaricaceae with bacterial community structure therefore suggest that dietary substrate identity may be more informative than dietary diversity alone. This interpretation is consistent with evidence that seasonal diets can outweigh host species in structuring the distal gut microbiota of yaks and Tibetan sheep (Wei et al. 2021).

The bacterial taxa distinguishing the groups provide mechanistic hypotheses rather than direct functional evidence. The greater representation of *Ruminococcus* and *Flavonifractor* in DS sheep is compatible with differences in the processing of complex plant polysaccharides and flavonoid‐derived substrates, whereas the AM‐associated assemblage may reflect a broader and nutritionally different substrate supply. These patterns accord with previous reports of coordinated microbial and metabolic variation in Tibetan sheep and other plateau herbivores (Gao, Chen, et al. 2025; Wei et al. 2021; Zhang et al. 2025). However, metabolic functions cannot be assigned confidently at the genus level because constituent species and strains may differ substantially. The PICRUSt2 findings require the same caution: predicted enrichment of transport and aromatic‐compound degradation in DS and glutamine/glutamate metabolism in AM is consistent with the dietary contrast but does not demonstrate pathway expression or metabolite flux. Shotgun metagenomics, metatranscriptomics and targeted functional assays are needed for verification.

### 4.3 Linking Dietary Plants, Faecal Bacteria and Metabolites

The metabolomic findings provide a chemical counterpart to the dietary and bacterial patterns. Enrichment of differential metabolites in flavonoid, flavone/flavonol and phenylpropanoid pathways is plausible under botanically distinct diets because many of these compounds originate from plants and are subsequently modified by host digestion and microbial reactions. Faecal abundance therefore integrates at least three processes: intake of plant compounds, host absorption or excretion and microbial transformation. Differences in phenolic acids and flavonoids between AM and DS should consequently be viewed as composite signatures of these processes rather than direct measures of microbial biosynthesis. The reciprocal relationship whereby phytochemicals alter microbial communities and microorganisms transform phytochemicals is consistent with previous work on diet–microbiome interactions (Tedeschi et al. 2021; Kiriyama and Nochi 2024; Ahsin et al. 2025).

Cross‐domain correlations support, but do not prove, this interpretation. Associations between plant families and phenolic metabolites are consistent with differences in the chemical substrates entering the gastrointestinal tract, whereas correlations between bacterial genera and the same metabolites may reflect microbial transformation, differential tolerance or shared responses to the forage environment. Similar studies have reported coordinated dietary, microbial and metabolic variation in plateau livestock and ungulates (Omondi et al. 2024; Zhang et al. 2025). The present study extends this framework by reconstructing the diets of free‐grazing Tibetan sheep and showing that plant‐family composition, rather than dietary diversity alone, was the principal correlate of bacterial differentiation. Because all components were measured concurrently, the direction of these relationships remains unresolved; controlled feeding or longitudinal studies are required to distinguish dietary substrate effects from microbial conversion and host metabolism.

### 4.4 Limitations and Implications

Several limitations define the scope of inference. Each grassland type was represented by one grazing area and one flock, and the six vegetation quadrats were spatial subsamples rather than independent grassland replicates. Grassland type is therefore confounded with site‐ and flock‐level differences, and the findings should be applied to the studied AM and DS areas rather than generalized to all northern Qiangtang grasslands. Sampling at one stage in late July also prevents separation of grassland‐associated variation from seasonal phenology or interannual conditions. In addition, faecal samples integrate processes across the gastrointestinal tract but cannot localize them to the rumen or hindgut; DNA metabarcoding is semi‐quantitative; and 16S‐based functional prediction and untargeted metabolite annotation require direct validation. Replicated multi‐site and longitudinal studies incorporating vegetation phytochemistry, digestibility, nutrient balance, rumen measurements and shotgun or transcript‐based functional analyses would provide stronger causal tests. Within these boundaries, the results indicate that assessments of grazing environments should consider botanical composition and phytochemical exposure alongside forage biomass and conventional nutrient indices.

## 5 Conclusions

In the studied grazing areas, Tibetan sheep in DS encountered lower forage biomass and nutrient concentrations but exhibited higher organic matter intake, lower dietary diversity and greater representation of Rosaceae than sheep in AM. These dietary differences were accompanied by distinct faecal bacterial communities and metabolite profiles. Plant‐family composition was more strongly associated with bacterial community variation than dietary alpha diversity, while the principal metabolic differences involved flavonoid‐ and phenylpropanoid‐related pathways. Predicted microbial functional differences were consistent with contrasting dietary substrate environments but require direct functional validation. Overall, the findings identify plant‐family identity and associated phytochemical exposure as potential links between forage conditions and faecal microbial–metabolic variation. Because the study compared one AM area and one DS area during a single sampling period, these findings represent coordinated associations rather than a demonstrated causal mechanism. They provide a basis for replicated longitudinal studies and suggest that botanical composition should be considered alongside forage biomass and conventional nutrient indices when evaluating Tibetan sheep grazing environments.

## Author Contributions

**He Ding:** formal analysis, investigation, visualization, writing – original draft. **Nan Liu:** resources. **Linyao Chen:** investigation. **Zenghui Liu:** investigation. **Wentao Zhang:** investigation. **Fangyu Gao:** investigation. **Kenan Li:** investigation. **Hai Wang:** investigation. **Jianshuang Wu:** conceptualization, funding acquisition, supervision, writing – review and editing. **Xiaoqing Zhang:** conceptualization, supervision, writing – review and editing.

## Funding

This work was supported by the Technology Program of Tibet Autonomous Region (XZ202501ZY0042) and the Key Research and Development and Conversion Program of Tibet Autonomous Region (XZ202601ZY0031).

## Conflicts of Interest

The authors declare no conflicts of interest.

## Supporting information


**Table S1:** Results of distance‐based redundancy analysis (db‐RDA) on alpha diversity indices and differential plants of diets.

## Data Availability

The raw faecal microbiota sequencing data have been deposited in the NCBI Sequence Read Archive (SRA) under BioProject accession number PRJNA1458458. The raw chloroplast trnL metabarcoding data used for dietary composition analysis have been deposited in the NCBI SRA under BioProject accession number PRJNA1458510.
