# Supplementary material for: Faecal Microbial and Metabolic Profiles Covary With Dietary Plant Composition in Tibetan Sheep Across Contrasting Grasslands
Source: Environ Microbiol Rep. 2026 Nov 9;18(6):e70443. doi: 10.1111/1758-2229.70443 (PMC13747824; doi:10.1111/1758-2229.70443)
Supplement: Supplementary file 1 [file EMI4-18-e70443-s001.docx]

Table S1. Results of distance-based redundancy analysis (db-RDA) on alpha diversity indices and differential plants of diets.

| Index | CAP1 | CAP2 | r2 | *P*_value |
| --- | --- | --- | --- | --- |
| Chao | -0.061 | -0.998 | 0.014 | 0.907 |
| Shannon | 0.729 | -0.684 | 0.068 | 0.612 |
| Pielou_e | 0.775 | -0.632 | 0.093 | 0.515 |
| Boraginaceae | 0.347 | -0.938 | 0.632 | 0.002 |
| Caryophyllaceae | -0.977 | 0.211 | 0.371 | 0.024 |
| Cyperaceae | -0.898 | 0.440 | 0.534 | 0.005 |
| Fabaceae | -0.999 | 0.036 | 0.196 | 0.105 |
| Mazaceae | -0.911 | 0.412 | 0.304 | 0.056 |
| Orobanchaceae | 0.396 | -0.918 | 0.126 | 0.372 |
| Plantaginaceae | -0.990 | 0.142 | 0.302 | 0.061 |
| Polygonaceae | -0.868 | 0.497 | 0.409 | 0.02 |
| Rosaceae | 0.966 | -0.258 | 0.929 | 0.001 |
| Tamaricaceae | -0.964 | 0.265 | 0.836 | 0.001 |
